# Malaria Incidence in Children in South-West Burkina Faso: Comparison of Active and Passive Case Detection Methods

**DOI:** 10.1371/journal.pone.0086936

**Published:** 2014-01-24

**Authors:** Alfred B. Tiono, David T. Kangoye, Andrea M. Rehman, Désiré G. Kargougou, Youssouf Kaboré, Amidou Diarra, Esperance Ouedraogo, Issa Nébié, Alphonse Ouédraogo, Brenda Okech, Paul Milligan, Sodiomon B. Sirima

**Affiliations:** 1 Centre National de Recherche et de Formation sur le Paludisme (CNRFP), Ouagadougou, Burkina Faso; 2 MRC Tropical Epidemiology Group, London School of Hygiene & Tropical Medicine (LSHTM), London, United Kingdom; 3 Serum Statens Institute, Copenhagen, Denmark; Mahidol-Oxford Tropical Medicine Research Unit, Thailand

## Abstract

**Background:**

The aim of this study was to determine the incidence and seasonal pattern of malaria in children in South-West Burkina Faso, and to compare, in a randomized trial, characteristics of cases detected by active and passive surveillance. This study also enabled the planning of a malaria vaccine trial.

**Methods:**

Households with young children, located within 5 kilometers of a health facility, were randomized to one of two malaria surveillance methods. In the first group, children were monitored actively. Each child was visited twice weekly; tympanic temperature was measured, and if the child had a fever or history of fever, a malaria rapid diagnostic test was performed and a blood smear collected. In the second group, children were monitored passively. The child’s parent or caregiver was asked to bring the child to the nearest clinic if he was unwell. Follow up lasted 13 months from September 2009.

**Results:**

Incidence of malaria (Fever with parasitaemia ≥5,000/µL) was 1.18 episodes/child/year in the active cohort and 0.89 in the passive cohort (rate ratio 1.32, 95% CI 1.13–1.54). Malaria cases in the passive cohort were more likely to have high grade fever; but parasite densities were similar in the two groups. Incidence was highly seasonal; when a specific case definition was used, about 60% of cases occurred within the 4 months June-September.

**Conclusion:**

Passive case detection required at least a 30%–40% increase in the sample size for vaccine trials, compared to active detection, to achieve the same power. However we did not find any evidence that parasite densities were higher with passive than with active detection. The incidence of malaria is highly seasonal and meets the WHO criteria for Seasonal Malaria Chemoprevention (SMC). At least half of the malaria cases in these children could potentially be prevented if SMC was effectively deployed.

## Introduction

Malaria remains a major public health problem in Burkina Faso. Malaria control policies include free distribution of insecticide treated bednets (ITNs), Intermittent Preventive Treatment (IPT) for pregnant women and treatment of malaria cases with artemisinin-based combination therapy (ACT). Seasonal Malaria Chemoprevention (SMC) was adopted as policy in 2012 and a national plan for introducing SMC has been prepared but has yet to be implemented. Coverage of ITNs and access to treatment with ACTs is relatively low [1] despite recent efforts in scaling up these interventions. Resistance to pyrethroids and DDT is now widespread in the country [2]–[4]. Parasitological confirmation of malaria is still not commonly used [5] with less than 20% of treatments based on a parasitological test [6]. National surveillance data are therefore of limited value and information about the incidence of malaria comes mainly from research studies. Recent studies in Boussé and Saponé districts in central Burkina Faso indicate that the burden of malaria remains very high with an average incidence of uncomplicated malaria ranging from 1.3 to 2.4 episodes of malaria per child per year [7], [8]. Little information is available about the burden in the south of the country.

Malaria surveillance relies essentially on two methods. Passive cases detection, whereby malaria cases are detected only at the clinic or by a village health worker when the child is brought for treatment, is generally now preferred over active case detection in many malaria intervention studies [9], but there have been few studies that have compared active and passive detection directly. Active surveillance, involves regular home visits at which the field worker checks the child’s health and tests for parasitaemia if the child has a fever or history of fever. It detects more malaria cases but is thought to have drawbacks for use in intervention trials. Early detection of cases may limit the scope to demonstrate efficacy against higher parasitaemias or more severe forms of disease; and treatment of any child with parasitaemia may bias assessments of efficacy which are usually based on a more specific case definition, due to the effects of post-treatment prophylaxis which may be unequal in the intervention groups. Use of a passive system in a clinical trial might give the best measure of impact that the intervention would have in reducing the burden on health facilities, but it underestimates the burden of malaria in the community as a whole.

Factors affecting the proportion of malaria morbidity detected passively include distance from health centre and treatment-seeking behavior [10].

As the trial has to be substantially larger if passive rather than active detection is used, it is surprising that few studies have compared active and passive detection directly. Snow *et al.*, 1989, in The Gambia [10], noted the large number of events which may be missed if frequent active follow-up is not performed. Olutu *et al.* (2010) in Kenya [11], found that incidence was three times as high with active compared to passive detection; they found both methods had high specificity provided fever was determined by measurement rather than reported history. We found only one randomized comparison, Ouedraogo *et al.* (2013) [8]. They compared the two methods in a randomized trial; incidence was higher in the active arm but they did not compare the severity of malaria cases.

The present study was conducted in the Cascades region in south-western Burkina Faso within an area selected for a phase 2b efficacy trial of the GMZ2 malaria vaccine candidate. The aim was to determine the incidence of malaria in this area, and to compare, in a randomized trial, characteristics of cases detected by active and passive surveillance; finally, the study aimed also to determine the burden and seasonal pattern of malaria disease in children.

Findings served to plan for sample size estimates and surveillance methods for the main GMZ2 malaria vaccine candidate trial.

## Methods

### Study Area

The study was conducted in Banfora Health District in the Cascades region, the south-western administrative area of Burkina Faso. The Cascades regional population in 2008 was estimated at 558,893 inhabitants of which 272,807 were residents in Banfora district [12] which covers a surface area of 15,871 km^2^. The climate is characterized by a rainy season from May to November and dry season from December to March (cold from December to February, hot from March to May). Malaria is the main cause of morbidity. Malaria transmission is stable throughout the year, but peaks during the rainy season (May through November). *Plasmodium falciparum* accounts for some 90% of malaria cases. The study area is served by two health facilities staffed by a minimum of three nurses each. Each health facility includes consultation room for sick visits, maternity ward and a pharmacy. These health facilities report to the Banfora health district hospital that is 5 km away.

### Recruitment of Study Participants

It was estimated that about 100 households minimum were required assuming an average of 7 children per household. This would give over 90% power to detect a difference in incidence if the incidence in the passive arm was less by 20% or more than the active arm; and if the incidence rate in the active arm was 1 episode per child per year assuming a coefficient of variation of 0.3 between households.

**Figure 1:**
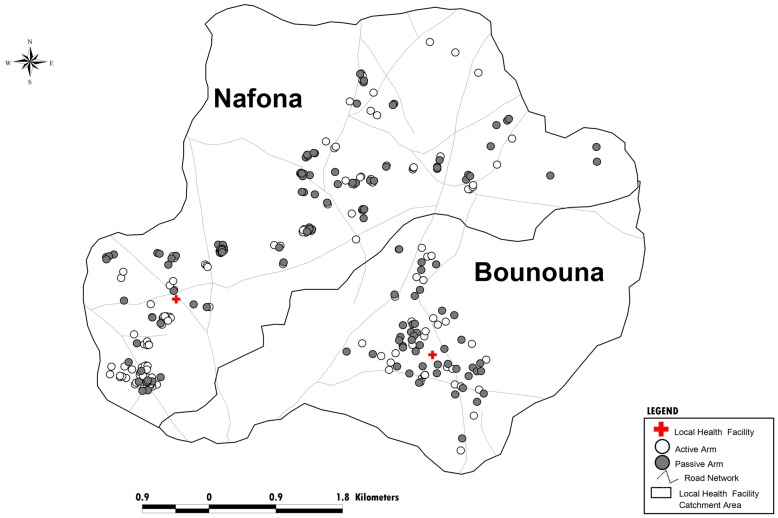
Map of the study area, Banfora district, Burkina Faso Households of study participants are identified as “white circle” when assigned to active case detection and “gray circle” when assigned to passive case detection.

In August 2009, prior to the start of the study, meetings were held in the villages in the study area to explain, in the local languages the objectives of the study and the procedures involved and to answer questions from the residents. In September 2009, eligible households (households were eligible if they were within an hour of the nearest health centre, had a child aged 0–5 and the child’s family planned to remain in the area for the one year period of the study) were randomly allocated to active case detection (ACD) or passive case detection (PCD) surveillance arms. Among the 271 household in the study area, 230 were eligible and therefore invited to participate to the random allocation process. This was done in a public lottery ceremony where heads of households were asked to participate to a traditional stick-drawing game. Sticks were white and red colors ended representing respectively the PCD and the ACD. The colored ends of the sticks were fixed in a box filled with sand so that they could not be seen by the participants. When a stick was drawn, the children in the household were assigned to the corresponding cohort. Neighboring households could be assigned to different surveillance methods (Figure 1). Children were enrolled during a baseline survey where caregivers from randomized households were invited to bring their children to the study team for assessment. Prior to any screening procedure, a written informed consent was obtained from parents or guardians. As per protocol, children with known HIV infection (HIV screening was not performed) or who were participating in another research study, or taking any prophylactic treatment against malaria were not included.

Each eligible child underwent a brief clinical examination by a physician, medical history was taken, and tympanic temperature measured. A finger-prick blood sample was obtained for measurement of haemoglobin using a hemocue®, (Ängelholm, Sweden), and for making malaria smears. A malaria rapid diagnosis test (RDT, Optimal-IT® DiaMed Basel, Switzerland) was performed for children with fever (axillary Temperature ≥38.0°C) and/or history of fever within the last 24 hours. Artemether-Lumefantrine (AL) was given to those with positive test as per national malaria treatment guidelines. If severe malaria was suspected the child was referred to the nearest health facility.

### Follow up of Study Participants

Follow-up of study participants took place from September 2009 until September 2010, a 13 months period. ID cards were provided to caregivers to facilitate recognition of the study participants by project staff. In the PCD cohort, parents/guardians of children were encouraged to take their child to the nearest health centre any time the child became unwell. Trained nurses were based at both health facilities in the study area to identify children in the trial and to ensure that they were seen, properly investigated in a standardized manner and treated appropriately. Local key informants in each village were requested to inform the study team when a child moved out of the study area or if a child died.

Parents/guardians of children in the ACD cohort were also encouraged to take their child to the clinic at any time they were sick. In addition, children in the ACD cohort were visited by trained field workers (qualified nurses) twice each week. At each visit the field worker administered a standardized questionnaire to the parents/guardians of the child to collect information about any illness since the last visit, symptoms experienced, use of healthcare facilities and any medications.

At each home visit in the ACD cohort, and whenever a child from either cohort attended clinic, tympanic temperature was measured using a digital thermometer. If the child had a fever (tympanic temperature ≥38.0°C), or history of recent fever (within the last 24 hours), a RDT (Optimal-IT® DiaMed Basel, Switzerland) for diagnosis of malaria was used to guide treatment and a thick blood smear was obtained for malaria microscopy. Children with a positive RDT were treated with AL for malaria based on National Malaria Control Program guidelines.

### Laboratory Investigations

Thick and thin blood films were prepared, air-dried, giemsa-stained, and examined using a light microscope fitted with 100X oil immersion lens. The number of parasites and leucocytes were counted to reach 200 leucocytes for positive slides. Slides were declared negative only after 100 high power fields had been read. Each slide was read twice by two independent microscopists. A third reading was performed in case of discrepancy between the two. Discrepancy of reading was defined as following: The ratio of densities from the first two readings is >1.5 or <0.67; or <30 parasites were counted with an absolute difference in the number of parasites >10; or discordance in positive-negative or species. The final result was based on the two most concordant readings. The parasitaemia was estimated on the basis of an assumed white cell count of 8,000/µL.

Microscopists competency was evaluated through an external quality control (EQC) program carried out by the College of American Pathology proficiency testing. It includes a set of 20 slides provided to each microscopist for reading thrice a year. Only those with a score of at least 80%, graded as ‘competent’, were involved in the reading of study participants’ slides.

### Ethics Statement

In adherence to the Council for International Organizations of Medical Sciences (CIOMS) guidelines, community permission was obtained before the start of the study. Written informed consent was obtained from parents/guardians of children who participated in the study. The study was approved by the National Ethical Committee of Burkina Faso (Deliberation N° 2009-36).

### Data Management and Statistical Methods

Data were reviewed for completeness and consistency then double entered into a Microsoft Access® database. The validated database was transferred to Stata 12.1 (College Station, Texas, USA) for analysis. The primary endpoint was malaria defined as the presence of fever (tympanic temperature ≥38°C) with a parasite density of 5,000/µL or more. Seven other case definitions were also considered (see Table 1).

**Table 1:** Cases definitions of malaria episodes.

Case definition	Description
**Primary outcome**	Documented fever with parasitaemia of 5,000/µL or more
**1**	Documented fever without obvious non-malaria cause
**2**	Documented fever or a history of fever in the last 24 hours without obvious non-malaria cause
**3**	Fever or history of fever with a positive RDT
**4**	Fever or history of fever with a positive blood film
**5**	Fever or history of fever with parasite density of 5,000/µL or more
**6**	Documented fever with a positive blood film
**7**	Documented fever with parasitaemia of 20,000/µL or more

The incidence rate of malaria was estimated for each case definition, including all malaria episodes that met the definition. All children were included in the denominator without deducting any time at risk after treatment. In the PCD cohort, observations were censored if the child was known to have left the study area or died, in the ACD cohort observations were censored if the child was lost to follow-up or died. In the primary analysis, any episodes occurring within 14 days of a primary episode which met the same case definition, were assumed to be relapses and did not contribute towards incidence. All children who were enrolled and followed up were included in the analyses. Jackknife confidence intervals using the household as the cluster were generated for incidence rates to allow for the lack of independence among multiple events in the same household. The Kaplan-Meier failure function was used to estimate the cumulative risk (the proportion of children with at least one episode of clinical malaria of each case definition) by the end of surveillance period, and the Nelson-Aalen cumulative hazard was used to estimate the mean number of episodes per child.

Cox regression was used to compare incidence rates in the ACD and PCD cohorts, using the household as the unit of randomization with a robust standard error to allow for the lack of independence among multiple events in the same household. The time scale used for the Cox regression was age. A Poisson regression with robust standard errors was used to examine the effect of surveillance method on non-zero parasite densities. A logistic random effects model that accounted for household-level effects was used to examine the effect of surveillance method on the proportion of cases with non-zero parasite density that exceeded 39.0°C and the proportion with parasite density of more than 20,000/µL.

An examination of the effect of season used calendar time categorized into six month bands of December to May and June to November, and interaction between surveillance method and season was examined.

## Results

### Participant Characteristics and Follow-up

**Figure 2:**
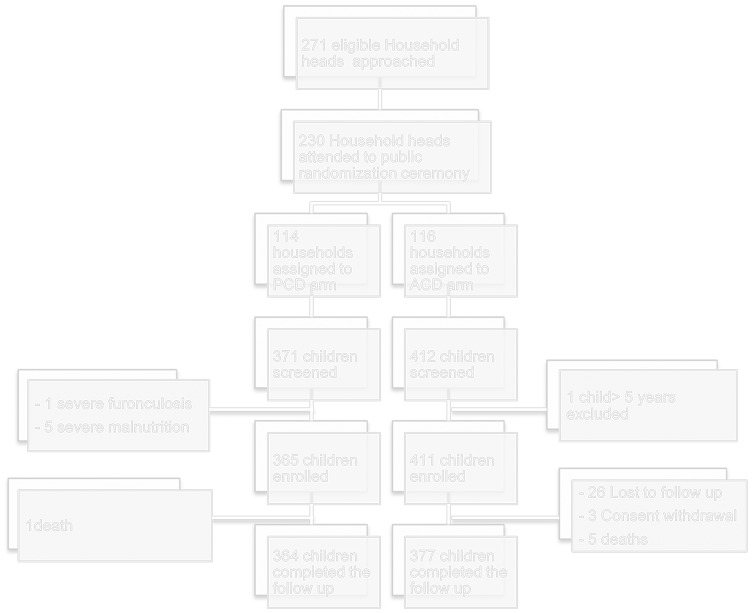
Study profile.

The study profile is presented in figure 2. During the baseline survey from 10–15^th^ September 2009, 698 children from 230 households were enrolled; 359 from 114 households were assigned to the PCD cohort and 339 from 116 households to the ACD cohort. Subsequently a further six children were enrolled in the PCD cohort upon attendance at a clinic and a further 72 children were enrolled in the ACD cohort after being identified at a household visit, from the randomized households (Table 2). Those enrolled late were similar to those enrolled during the baseline survey in terms of age or sex (Table 2). Baseline characteristics among PCD and ACD cohorts were approximately similar (Table 2).

**Table 2:** Characteristics of the study population, number and (percentage).

	Enrolled during baseline survey		Enrolled after baseline survey	
Characteristic	PCD (n = 359)	ACD (n = 339)	PCD (n = 6)	ACD (n = 72)
**Girls**	186 (52%)	145 (43%)	3 (50%)	31 (43%)
** Age <1 year**	55 (15%)	50 (15%)	2 (33%)	9 (13%)
** 1–<2 years**	101 (28%)	83 (24%)	1 (17%)	21 (29%)
** 2–<3 years**	68 (19%)	63 (19%)	2 (33%)	12 (17%)
** 3–<4 years**	73 (20%)	83 (24%)	1 (17%)	17 (24%)
** 4–<5 years**	62 (17%)	60 (18%)	0	13 (18%)
**Bednet used** *	194 (54%)	189 (56%)	Unknown	Unknown
**LLIN used** **	166 (46%)	167 (49%)	Unknown	Unknown
**Mean (SD) Haemoglobin** ***	9.26 (1.46)	9.03 (1.47)	Unknown	Unknown

* 3 children in the PCD and 1 child in the ACD cohorts had missing data for bednet use.

** 5 children in the PCD and 5 children in the ACD cohorts had missing data for LLIN use.

*** 5 children in PCD and 1 child in ACD had missing Haemoglobin.

In total, 29 children were known to be lost to follow-up or moved from the study area in the ACD cohort, with a median duration of 295 days (95% CI: 290–297) of follow-up time. Total follow-up was 421 person-years in the ACD cohort and 382 person-years in the PCD cohort.

In total ten children were admitted to hospital during the study period. Among them, three were from the ACD cohort and seven from the PCD cohorts. For children in the ACD cohort, the main cause was severe malaria (two cases of severe anemia and 1 case of prostration). In the PCD cohort, causes for admission were severe gastroenteritis (1 case), severe malaria (2 cases with convulsions and 1 case of respiratory distress), moderate anemia (1 case) and respiratory infection (1 case).

Six children died in the course of the study. In the ACD cohort, three boys aged one, one boy aged two and one girl aged four died; among them three occurred during the low transmission season (April) and two during the malaria high transmission season (in June and August). One girl aged one year from the PCD cohort died during the malaria transmission season. Cause of deaths is not known (none of the deaths occurred in hospital and verbal autopsy was not performed).

### Number of Visits

The 411 children in the ACD cohort contributed 40,490 visits, median 101 per child (IQR 99–102). Almost all of these children (388, 94%) attended the health clinic, with a median of 5 clinic visits (IQR 2–7) per child for a total of 1952 clinic visits. Of the 365 children in the PCD cohort, 329 (90%) contributed to 1286 clinic visits for a median of 3 visits (IQR 2–5) per child.

### Incidence of Clinical Malaria Episodes and Comparison of Passive and Active Surveillance

**Figure 3:**
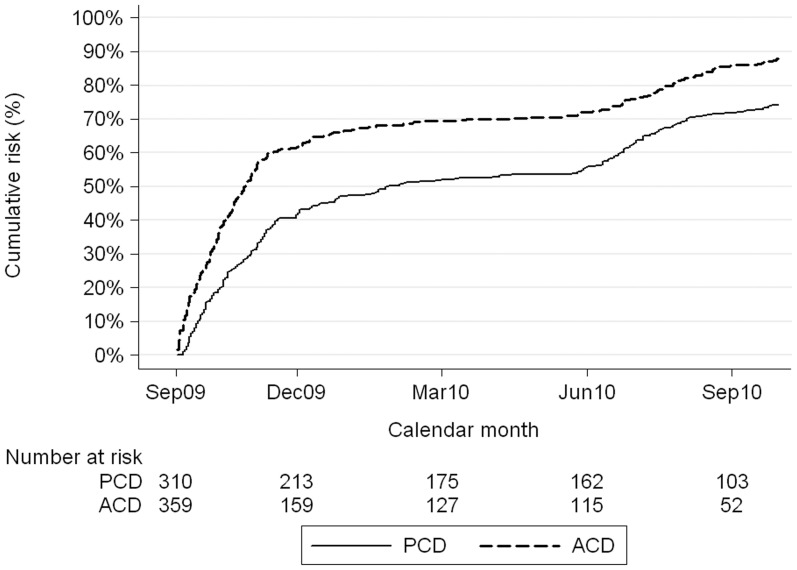
Nelson-Aalen cumulative hazard, mean number of children with a measured fever and a parasite density of 5,000/µL or more, by surveillance method.

- **Malaria with fever and parasitaemia 5,000/µL or more:** In the ACD cohort the number of episodes per child ranged from 0 to 5, with a mean of 1.18. In the PCD cohort, the mean number of episodes per child was 0.89 (range: 0 to 6). Timing of cases is shown in Figure 3. The percentage of children that remained free of malaria was 38% in the ACD and 46% in the PCD (Table 3). Incidence was higher in the rainy season May to November, with a peak from June to September, than in the dry season (see additional file S1). Incidence was higher in younger children (see additional file S2). There was no difference in incidence between LLINs users (Rate 1.08/person- year, 95% CI 0.96–1.23) and children who did not use LLINs (Rate 0.93/person-year, 95% CI 0.82–1.07). The percentage of annual cases (October 2009 to September 2010) that fell in the four months June to September 2010 was 59% (440/796 cases; Table 4). During the higher transmission season, in the months of June through November, incidence was 39% higher in the active surveillance group. During the low transmission season, December through May, incidence was similar in active and passive groups (Table 5).
- **Other case definitions of clinical malaria episodes:** A greater number of episodes of malaria were observed by active case detection compared to passive case detection for all case definitions (Table 3). The highest number of episodes of malaria was 11 episodes in one child recorded over the 13 months study period.

**Table 3:** Number (and range) of episodes of malaria per child, and proportion of children remaining malaria free, by surveillance method.

	Number (Range) of episodes		Proportion of children remaining malaria free	
Case definition	PCD cohort	ACD cohort	PCD cohort	ACD cohort
**Primary outcome**	0.89 (0–6)	1.18 (0–5)	46%	38%
**1**	1.53 (0–7)	1.98 (0–9)	27%	18%
**2**	2.43 (0–8)	3.85 (0–11)	14%	5%
**3**	1.47 (0–6)	2.25 (0–8)	26%	12%
**4**	1.61 (0–7)	2.41 (0–8)	22%	12%
**5**	1.28 (0–6)	1.84 (0–6)	32%	23%
**6**	1.10 (0–6)	1.44 (0–6)	37%	29%
**7**	0.69 (0–6)	0.93 (0–5)	55%	45%

**Table 4:** Number and proportion of the annual* malaria cases falling in the four months June to September 2010.

Case definition	PCD cohort	ACD cohort	All children
**Primary outcome**	174/328 (56%)	266/468 (62%)	440/796 (59%)
**1**	238/559 (45%)	374/789 (52%)	612/1348 (49%)
**2**	324/887 (39%)	605/1542 (44%)	929/2429 (42%)
**3**	251/536 (51%)	440/898 (55%)	691/1434 (53%)
**4**	258/594 (46%)	460/961 (53%)	718/1555 (50%)
**5**	226/471 (51%)	375/735 (56%)	601/1206 (54%)
**6**	194/403 (51%)	308/569 (59%)	502/972 (56%)
**7**	145/252 (60%)	221/368 (64%)	366/620 (63%)

* The year studied was from 1^st^ October 2009 to 30^th^ September 2010.

**Table 5:** Incidence rate ratios for active versus passive detection.

Case definition	Crude RR (ACD:PCD) (95% CI)	Season	RR stratified by season*	p-value for interaction
Primary outcome	1.32 (1.13,1.54)	Dec–May	1.03 (0.70,1.52)	0.15
		Jun–Nov	1.39 (1.18,1.63)	
1	1.31 (1.17,1.47)	Dec–May	1.02 (0.83,1.25)	0.002
		Jun–Nov	1.45 (1.28,1.66)	
2	1.61 (1.47,1.77)	Dec–May	1.24 (1.08,1.43)	<0.001
		Jun–Nov	1.83 (1.66,2.02)	
3	1.54 (1.38,1.73)	Dec–May	1.11 (0.87,1.41)	0.001
		Jun–Nov	1.68 (1.49,1.89)	
4	1.49 (1.34,1.66)	Dec–May	1.07 (0.85,1.34)	<0.001
		Jun–Nov	1.62 (1.44,1.82)	
5	1.44 (1.27,1.63)	Dec–May	1.15 (0.86,1.54)	0.08
		Jun–Nov	1.52 (1.33,1.73)	
6	1.30 (1.14,1.49)	Dec–May	0.94 (0.69,1.28)	0.02
		Jun–Nov	1.39 (1.15,1.66)	
7	1.35 (1.13,1.62)	Dec–May	1.14 (0.72,1.79)	0.36
		Jun–Nov	1.41 (1.17,1.70)	

**Figure 4:**
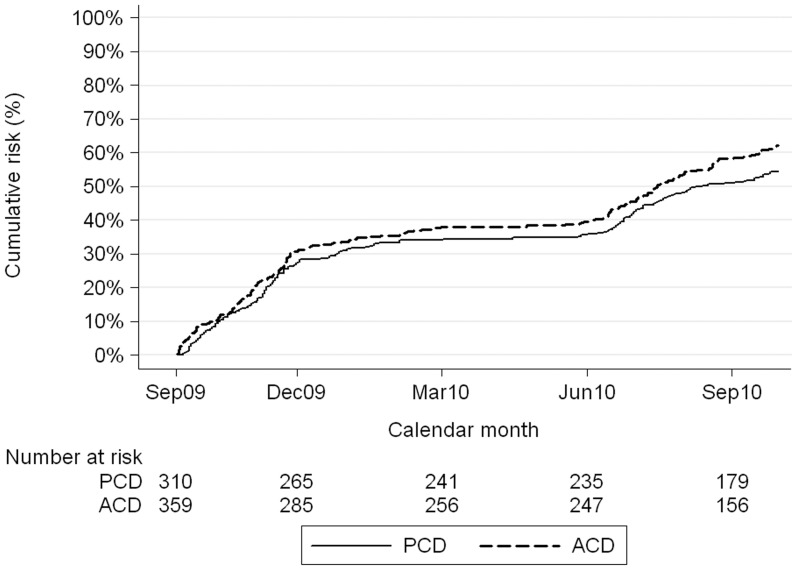
Nelson-Aalen cumulative hazard, mean number of children with a measured fever or history of fever in the last 24 hours and a positive RDT, by surveillance method.

The percentage of annual cases (October 2009 to September 2010) that fell in the four months June to September 2010 ranged from 39% to 64% depending on the case definition used and the type of surveillance; the higher figure corresponding to the more specific malaria case definitions (Table 4). For the majority of case definitions, there was a significant interaction between the effect of surveillance method and season (Table 5). At least 39% more malaria cases were identified by active surveillance than passive surveillance during the months of June to November (Table 5). The majority of cases occurred during these months; for example children with documented or history of fever and a positive RDT (Figure 4). In comparison, there was little evidence of a difference in the cases detected by passive or active surveillance during the months December to May.

### Grade of Fever and Parasite Density among Malaria Cases Presenting with Parasites

**Figure 5:**
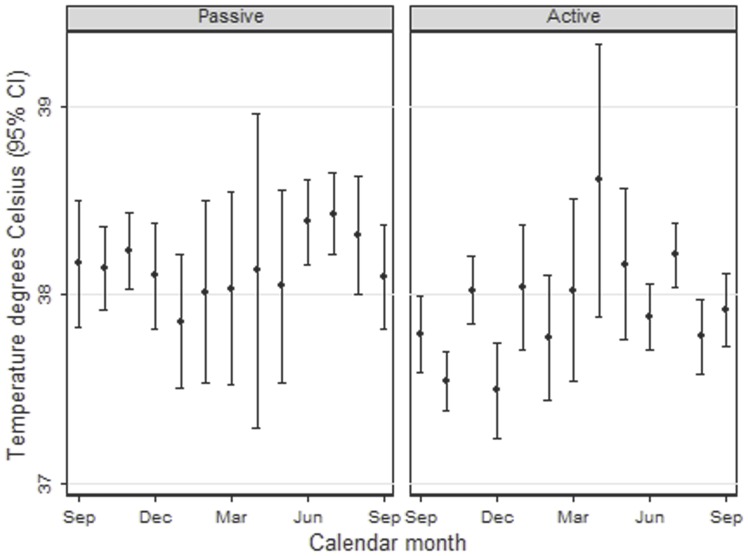
Arithmetic mean tympanic temperature (°C) among children with parasite positive malaria cases by calendar month.

The proportion of clinical malaria cases presenting with a high grade fever (≥39.0°C) was higher in the PCD cohort (26%) than the ACD cohort (19%) (Figure 5), this effect varied with season (interaction P-value p = 0.05), the odds ratio for presenting with a high grade fever in the PCD cohort compared to the ACD cohort during the months of June to November was 1.72 (95% CI: 1.3–2.2).

**Figure 6:**
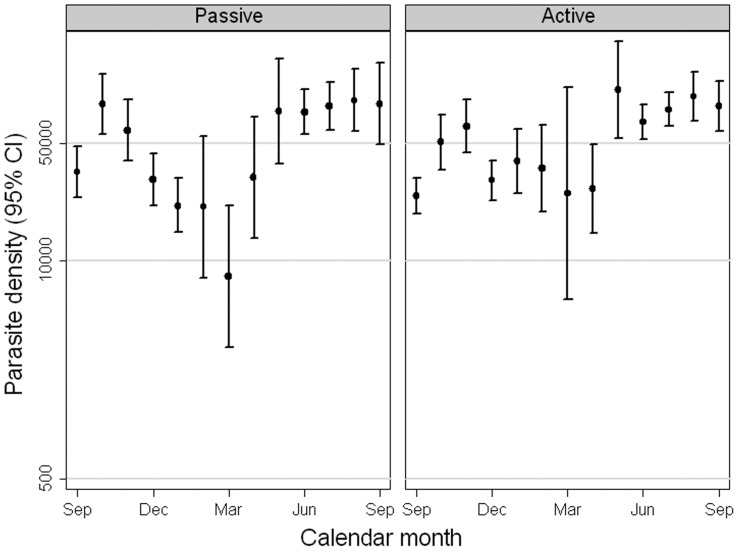
Arithmetic mean parasite density among parasite positive malaria cases by calendar month.

Parasite density varied by season (Figure 6). Mean parasite density among malaria cases (fever or history of fever with any parasitaemia by microscopy) was similar among cases in PCD cohort than in the ACD cohort. During the wet season the mean was 75,261 for June to November in the PCD cohort versus 65,997 in the ACD cohort (ratio ACD/PCD: 0.88, 95% CI 0.72–1.07). The proportion of clinical malaria cases with high parasite density, above 20,000, was also similar (63% in the PCD cohort and 58% in the ACD cohort, OR 0.80, 95% CI: 0.62–1.03) from June to November.

### Association between Parasite Density and the Presence of Fever at Baseline

**Figure 7:**
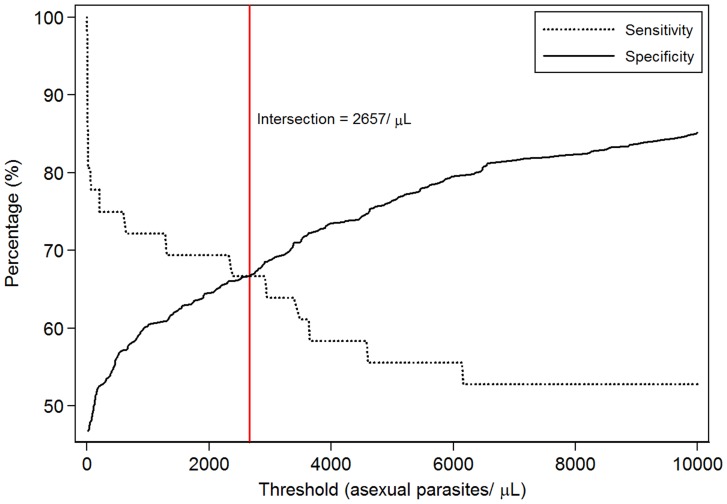
Malaria case definition using objective fever (Temperature≥38.0): Sensitivity and specificity of alternatives parasite threshold.

**Figure 8:**
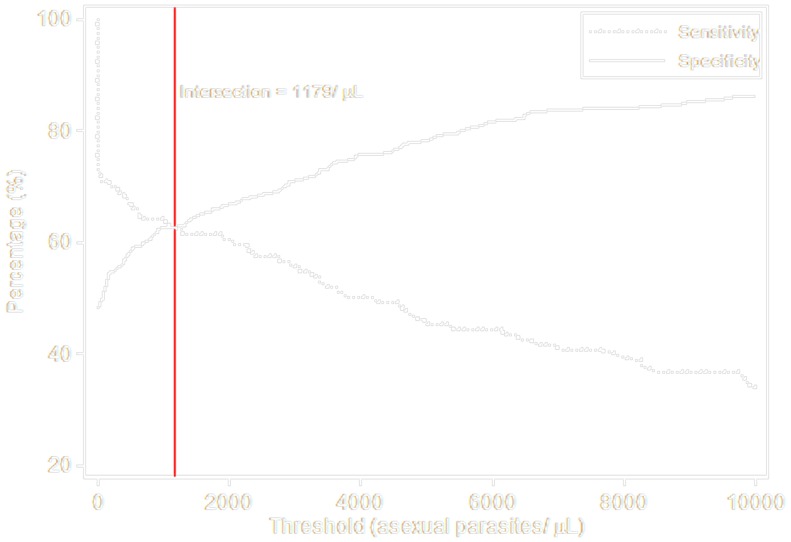
Malaria case definition using objective fever (Temperature≥38.0)/History of fever: Sensitivity and specificity of alternatives parasite threshold.

ROC curves were produced showing sensitivity and specificity of parasite density cut-offs for identifying malaria cases (parasitaemia with tympanic temperature ≥38.0°C or parasiteamia with tympanic temperature ≥38.0°C/History of fever within the last 24 hours), using data collected at the baseline survey (Figures 7 and 8). If fever only was considered, a cut-off of 5,000/µL had sensitivity of 56% and specificity of 76%. If both fever and/or history of fever were considered, the sensitivity and specificity were respectively 48% and 78% at the threshold of 5,000/µL.

### Accuracy of Malaria Rapid Diagnosis Test

Specificity of Optimal-IT® is summarized in Table 6. Overall the specificity was 88% in the PCD cohort vs. 90% in the AC cohort. Lowest specificity was recorded during the peak of malaria transmission season (July to September) in both cohorts.

**Table 6:** Specificity of RDT in relation to malaria cases (>0 parasites/µL) by calendar month.

	PCD cohort	ACD cohort	All children
**Calendar month**			
**Sep 2009**	83%	98%	94%
**Oct 2009**	94%	87%	89%
**Nov 2009**	88%	88%	88%
**Dec 2009**	84%	91%	89%
**Jan 2010**	92%	100%	96%
**Feb 2010**	96%	93%	94%
**Mar 2010**	86%	94%	90%
**Apr 2010**	85%	86%	86%
**May 2010**	95%	93%	94%
**Jun 2010**	86%	91%	90%
**Jul 2010**	71%	69%	70%
**Aug 2010**	80%	63%	69%
**Sep 2010**	77%	91%	86%
**Overall**	**88%**	**90%**	**89%**

## Discussion

Malaria remains the main cause of severe illness and death among children in Burkina Faso. Our study confirms that incidence remains very high despite recent efforts to scale-up the use of LLINs and access to ACTs, with an average incidence of over 2 episodes of malaria confirmed by RDT per child per year. Incidence was high even among children reported to be using LLINs. Coverage of LLINs was only 50% in the children in this study, we did not measure coverage in other age groups in the population, but it is likely to be lower. The overall effect of LLINs may be greater if higher levels of community coverage can be achieved. Resistance to pyrethroids, known to be common in southern Burkina Faso, has become widespread in the country in recent years [2] but the impact of this on the personal protection from using an LLINs is unclear.

Malaria morbidity showed marked seasonal variation, with higher incidence during the rainy months of June-November. The degree of seasonality was underestimated when less specific case definitions were used; using a specific case definition (documented fever with parasites density 5,000/µL or more), 62% of annual cases occurred in 4 months of the year. The WHO now recommends that children living in areas of the Sahel sub region with highly seasonal malaria, defined as 60% of cases falling in 4 months of the year benefit from seasonal malaria chemoprevention (SMC) [13]. Our data indicate that South-Western Burkina Faso is one of the areas where SMC should be introduced.

This study was conducted in the context of field site characterization to prepare for a phase IIb efficacy trial of the GMZ2 malaria vaccine candidate [14]–[16], and to pilot PCD and ACD surveillance methods. Passive detection has been preferred for vaccine trials due to a concern that active surveillance, with early detection and treatment of cases, creates an artificial situation that may interfere with assessment of vaccine efficacy. For evaluation of blood stage vaccines, which seek to reduce the severity of infections by limiting parasites density, detecting cases before parasitaemia has fully developed may limit the ability to detect an effect of the vaccine. However, although passively detected cases were more likely to have high grade fever, we found no evidence that parasite densities were lower with active surveillance, among cases with documented fever.

Incidence of malaria was substantially lower using passive detection through clinics, even though all households had good access to clinic. For our primary endpoint of documented fever with parasitaemia of 5,000/µL or above, the rate ratio ACD:PCD during the main transmission period was 1.39; so PCD would require a 39% increase in the sample size for a vaccine efficacy trial, compared to ACD.

Strength of our study is that it was randomized by household, and sample size was relatively large (230 households enrolled while minimum sample size required was 100 households). The location of participants could be a confounder for comparison between surveillance methods given the known heterogeneity in malaria exposure [17]. The randomization by household minimized the risk of confounding due to differences in malaria exposure or access to health care. Ouedraogo *et al.* also compared active and passive surveillance in a randomized study where randomization was done by village, but they did not consider differences in clinical characteristics of the cases.

Occurrence of clinical malaria is the endpoint most commonly used in the field to measure not only the efficacy of interventions to prevent malaria but also to assess the public health burden of the disease. However it is not always straightforward to clearly define what constitutes an episode of clinical malaria especially in areas of high malaria endemicity where asymptomatic carriage of *P. falciparum* infection is common. The cut-off of parasite density that gives same level of sensitivity and specificity is comparable to what was reported in other part of Burkina Faso [8] and other west African countries with similar malaria transmission pattern [18], [19].

The specificity of testing by Optimal RDT in our study was lower during the malaria high transmission season. It is possible that in our context with malaria high endemicity, a high proportion of individuals are carrying low parasites density not detected by microscopy despite the experience of microscopists and the quality control using double reading of each individual blood smear.

It is likely to be many years before a highly effective malaria vaccine is available. Our study shows that malaria burden in young children remains unacceptably high in South West Burkina Faso despite recent efforts to improve access to insecticide treated bednets and effective treatment with ACTs. Alternative measures are urgently needed. We have shown that malaria incidence is highly seasonal in South West Burkina Faso making this area suitable for SMC. Over 60% of malaria cases each year could potentially be prevented if SMC was provided over 4 months of the year from July to October.

## Supporting Information

File S1 **Monthly incidence rates of malaria by calendar month, September 2009 through to September 2010.** Monthly incidence rates are presented for all eight case definitions of malaria. A breakdown is provided for children in the passive and active case detection cohorts as well as incidence rates for all children under follow-up. Rates are presented in both tabular and graphical format. In addition, the proportion of cases (from the full calendar year October 2009 to September 2010) falling in the four months of June to September 2010 is provided.
(XLSX)

File S2 **Incidence rates of malaria by yearly age groups, September 2009 through to September 2010.** Yearly incidence rates are presented for all eight case definitions of malaria. A breakdown is provided for children in the passive and active case detection cohorts as well as incidence rates for all children under follow-up. Rates are presented in both tabular formats.
(XLSX)
